# High variability in the measurement of HIV primary prevention activities and outcomes

**DOI:** 10.1002/jia2.25645

**Published:** 2020-12-20

**Authors:** Sayuri Sekimitsu, Jacqueline DePasse, Michelle Morrison, Mary Mahy, Brian Rice, Kristen Earle, Kate Daley, Jim Larson, Anna Carter, Geoff P Garnett, Charles B Holmes

**Affiliations:** ^1^ Boston Consulting Group Boston MA USA; ^2^ Bill & Melinda Gates Foundation Seattle WA USA; ^3^ UNAIDS Geneva Switzerland; ^4^ London School of Hygiene & Tropical Medicine London UK; ^5^ Georgetown University Washington DC USA; ^6^ Johns Hopkins University School of Medicine Baltimore MD USA

**Keywords:** HIV prevention, HIV, AIDS, primary prevention, monitoring and evaluation, indicators

## Abstract

**Introduction:**

While there is a global consensus on monitoring Human Immunodeficiency Virus (HIV) treatment progress, there has been less attention to the degree of consistency of the measurement of HIV prevention programmes—and the global prevention response is not on‐track to achieve 2020 goals. In this paper, we assess the degree of variability in primary prevention indicators selected by national strategic plans (NSPs) and global stakeholder monitoring and evaluation (M&E) strategies.

**Methods:**

We obtained the most recent NSPs from low and middle income Joint United Nations Programme on HIV/AIDS (UNAIDS) Fast‐Track countries, and M&E documents from The Global Fund to Fight AIDS, Tuberculosis and Malaria (The Global Fund), President’s Emergency Plan for AIDS Relief (PEPFAR), UNAIDS, the Global HIV Prevention Coalition and the World Health Organization (WHO). We extracted HIV primary prevention indicators from each document, standardized and aggregated them by age/ sex, categorized indicators by topic, and evaluated the frequency of matched indicators between countries and stakeholders. Data were collected between February and April of 2019.

**Results:**

Twenty‐one NSPs and five global stakeholder documents were assessed; 736 primary prevention indicators were identified; 284 remained following standardization and aggregation. NSPs contained from 3 to 48 primary prevention indicators, with an average of 23; categories included: HIV education and outreach (17.6%), testing (17.3%) and condom use (16.2%). Of unique national indicators, only 34% was shared between two or more countries. Sixty‐nine per cent was applied in a single country only. 56% of NSP indicators did not appear in any global stakeholder document. Conversely, 42% of global indicators did not appear in any surveyed NSPs. Within global indicators, 63% was only measured by one global body, and no single indicator was measured by all five.

**Conclusions:**

These analyses reveal a lack of consensus both between and within countries’ and global stakeholders' measurement of HIV prevention. Though some variability is expected, these findings point to a need to refocus attention on achieving greater consensus on a global measurement framework for HIV prevention.

## 1 INTRODUCTION

Substantial progress has been made in responding to the HIV epidemic over the past decade, with new HIV infections reduced by an estimated 40% since their peak in 1996, and a 60% decrease in Human Immunodeficiency Virus/Acquired Immunodeficiency Syndrome (HIV/AIDS)‐related mortality since 2005 [1]. However, HIV remains a global public health threat. An estimated 1.7 million people became newly infected with HIV in 2019, far above the United Nations General Assembly goal of fewer than 500,000 new HIV infections in 2020 [1, 2].

In recent years, there has been a focus on expanding treatment access to improve patient outcomes and decrease the risk of onwards transmission; however, in some settings, there has been insufficient attention to primary HIV prevention. In 2012, after preventive benefits of HIV treatment were confirmed by the HPTN052 trial, models projected that HIV incidence would decrease by 35% to 54% within eight years in areas where population coverage levels reached 80% [3]. However, declines of this magnitude have not been observed in studies on a population level in the test and treat trials, highlighting the continued need for evidence‐based combination prevention interventions [4, 5, 6].

One of the strengths of the treatment response is the clarity of programmatic goals and indicators. The 2014 UNAIDS 90‐90‐90 targets (90% of all people living with HIV know their status, 90% of all people with diagnosed HIV infection will receive sustained antiretroviral therapy and 90% of all people receiving antiretroviral therapy will have viral suppression by 2020) helped to streamline and prioritize monitoring and evaluation efforts for treatment and arguably served as an effective and powerful rallying and advocacy tool [7]. There is strong global alignment behind these targets, from the U.S. President’s Emergency Plan for AIDS Relief (PEPFAR), Joint United Nations Programme on HIV/AIDS (UNAIDS), to The Global Fund to Fight AIDS, Tuberculosis and Malaria and the World Health Organization (WHO) [8, 9, 10, 11, 12]. Having a clear, focused treatment target has mobilized and influenced these decision makers, with substantial progress made in achieving the targets since their launch [13, 14].

Although there have been efforts to establish clear prevention goals, such as the establishment of UNAIDS’s five pillars [2], national governments and global stakeholders all have processes to produce monitoring and evaluation indicators [10, 12]. There has been limited examination of the range of prevention‐related indicators in use and the degree to which global stakeholders and governments are aligned in their measurement. Without this understanding, it is difficult to understand the feasibility and path forward to attain more streamlined indicators and global targets.

In order to understand the degree of consensus in HIV primary prevention measurement and evaluation, we review the variability in the primary prevention indicators selected in the country national strategic plans (NSPs) and global monitoring and evaluation documents. In addition, we examine the frequency of categories of primary prevention indicators used most consistently across stakeholders.

## 2 METHODS

### 2.1 Data collection

#### 2.1.1 Data collection in country NSPs

Countries were included for analysis if they were one of the 28 low/middle‐income countries (LMIC) included in the UNAIDS Fast Track list (Table 1) [15]. The 2015 UNAIDS Fast Track list countries were selected for their need to accelerate their HIV response in order to meet the global target of ending AIDS by 2030. The 28 low/middle‐income countries and two high‐income countries on the Fast Track list account for 89% of all new HIV infections [15]. This analysis included only the low‐ and middle‐income countries. The search strategy for the NSP documents included an internet search of the country name, “HIV” and “National Strategic Plan” or “National framework” or “Monitoring and Evaluation framework,” and the Ministry of Health website for each country. Representatives of each country’s Ministry of Health were contacted through publicly available contact information found on the website. Data were collected between February and April of 2019. The most recent publicly available NSPs were compiled for each country. Any NSP that was published and applied (i.e. years in scope) prior to 2014 were excluded. Indicators were identified within each NSP by searching for tables containing the term “indicator”; these were recorded and subsequently evaluated based on inclusion/exclusion criteria.

**Table 1:** Inclusion and aggregatiaon criteria

Inclusion criteria	Either all of the following: Indicator is in a table labeled with the term "indicator" Indicator is associated with HIV acquisition and incidence (including discrimination against people living with HIV and sero‐discordant couples) Indicator is in "activity," "output," and "outcome" category of data collection^a^ Or, document categorizes indicator as a HIV prevention indicator
Exclusion criteria	Indicator is associated with mother‐to‐child transmission Indicator is in "assessment and planning," "inputs," or "impacts" category of data collection^a^
Aggregation criteria	Indicators were aggregated by: Age Sex Percent vs. number vs. percent/number Indicators remained disaggregated by: Key populations: men who have sex with men, people who inject drugs, sex workers, transgender people, prisoners Priority populations: adolescent girls and young women, mobile populations, non‐injecting drug users, military Other distinct populations: vulnerable populations, pregnant/breastfeeding women, sexual violence victims, people living with HIV

^a^ Inputs – financial, human, or material resources used in a programme or intervention; Activities – actions taken or work performed through which inputs are mobilized to produce outputs; Outputs – intermediate effects of an intervention's outputs, such as change in knowledge, attitudes, beliefs, behaviors; Impacts – the long‐term, cumulative effects of programmes or interventions.

#### 2.1.2 Selection of global bodies

Consultations with experts in the field of HIV prevention identified the following global monitoring and evaluation documents for review: The Global Fund HIV Monitoring & Evaluation Framework (GF M&E) (2017), PEPFAR Monitoring, Evaluation and Reporting (MER 2.0) Indicator Reference Guide (2017), UNAIDS Global AIDS Monitoring (GAM) (2018), UNAIDS Global HIV Prevention Coalition Indicators (PC) (2017) and WHO Consolidated Strategic Information (SI) Guidelines (2015) [8, 9, 10, 11, 12]. These documents were obtained from each of the global organizations’ websites.

### 2.2 Inclusion/exclusion criteria for indicators

Each indicator within each NSP and global stakeholder document was reviewed and evaluated based on predefined inclusion/exclusion criteria that are detailed in Table 1. All indicators relevant to the primary prevention of incident HIV infections were included. Indicators related to antiretroviral treatment and prevention of mother to child transmission (PMTCT) were excluded. HIV testing indicators were included in the analysis because HIV testing is a necessary pre‐condition for eligibility for most HIV prevention interventions, and serves as the entry point to the prevention cascade.

Each indicator included in our study was categorized within a measurement and evaluation indicator logical framework [16]. Indicators were included if they were defined as “activities,” “outputs,” or “outcomes,” and excluded if defined as “assessment and planning,” “inputs,” or “impacts” to capture indicators relevant to primary prevention [16].

### 2.3 Data extraction and indicator standardization and aggregation

For each indicator, population type, age group (if available), numerator and denominator was extracted and recorded. These fields were then standardized across country NSP indicator lists and global indicator lists on these fields, such that exact language was not necessary to constitute a match. For example “men who receive voluntary male medical circumcision” and “men who undergo voluntary male medical circumcision,” would be standardized to “men who receive voluntary male medical circumcision.” Indicator language standardization was performed by SS and confirmed by JD.

Following extraction and standardization from the reviewed documents, indicators were aggregated by age and sex, to minimize over‐reporting of variability based on minor variation in disaggregation of age and sex amongst nations and global stakeholders. They were also combined if they measured “percent of” or “number of”. For example if one country measured “% of men aged 15 to 20 who are circumcised” and one global body measured “# of men aged 30 to 39 who are circumcised” this would be considered a match between the country and global body. However, indicators remained disaggregated by key populations (defined groups who are at increased risk of HIV, in any context), priority populations (groups who are at increased risk of HIV, in certain contexts) and other distinct populations (Table 2).

### 2.4 Data analysis

The indicators collected by each country were identified. For each country, indicators were evaluated by whether they appeared in any global stakeholder monitoring and evaluation (M&E) document. The proportion of indicators not in any evaluated global stakeholder M&E documents was calculated, by country and averaged across countries. The indicators listed by countries were evaluated by whether they were present in any global stakeholder prevention indicator set and the number of country NSPs. We conducted sub‐analyses to examine variability in NSP indicators within the high burden region of Eastern and Southern Africa [17], and by countries with high vs. low burdens (countries with “high” burdens have >1% prevalence of HIV and countries with “low” burdens have <1% prevalence of HIV) [18]. We also compared HIV prevalence [18] to the number of indicators in a country NSP and calculated a Pearson’s correlation coefficient (r).

Similarly, the number of indicators identified by each global stakeholder were compiled. Each global indicator was evaluated by whether it was documented in any country NSP. For each indicator, the number of countries that identified the indicator, as well as the number of global stakeholders that identified the indicator was assessed. The authors used Microsoft Excel (version 10) to house and analyse the extracted data.

## 3 RESULTS

Of the 28 2015 LMIC Fast‐Track countries, the NSP for 24 (86%) were obtained (Table 2a). Of these, seven were translated to English using online translation services: Angola (Portuguese), Cameroon (French), Chad (French), China (Mandarin), Cote d’Ivoire (French), Ukraine (Ukrainian) and Vietnam (Vietnamese). The NSPs for Jamaica, Vietnam and China did not include any indicators, so these three countries were also excluded from the analysis leaving 21 countries to be included. Documents were also evaluated for five global stakeholders (Table 2b). In total, 736 unique primary prevention indicators were extracted. After standardization and aggregation, 284 unique primary prevention indicators remained for analysis.

**Table 2a:** Number of indicators in included low/middle income Fast‐Track countries

Country	Years covered in national strategic plan	Total number of indicators	Number of excluded indicators	Number of included indicators
Angola	2015 to 2018	67	34	33
Cameroon	2014 to 2017	47	25	22
Chad	2012 to 2015	163	79	84
Cote d'Ivoire	2016 to 2020	105	62	43
Democratic Republic of Congo	2014 to 2017	60	23	37
Ethiopia	2015 to 2020	23	9	14
India	2017 to 2024	61	41	20
Indonesia	2010 to 2014	29	11	18
Kenya	2014 to 2019	120	74	46
Lesotho	2014 to 2018	64	18	46
Malawi	2015 to 2020	70	36	34
Mozambique	2016	12	7	5
Nigeria	2017 to 2021	48	16	32
Pakistan	2015 to 2020	108	20	88
South Africa	2017 to 2022	104	47	57
Swaziland	2014 to 2018	96	39	57
Tanzania	2016 to 2018	74	46	28
Ukraine	2014 to 2018	28	17	11
Uganda	2015 to 2020	72	41	31
Zambia	2017 to 2021	69	37	32
Zimbabwe	2015 to 2018	27	13	14

Indicator count refers to indicators as listed on original document.

**Table 2b:** Number of indicators in included key global stakeholder documents

Key global stakeholder	Year published	Total number of indicators	Number of excluded indicators	Number of included indicators
UNAIDS GAM^a^	2018	143	22	121
Global Fund M&E^b^	2017	75	25	50
PEPFAR MER^c^	2017	83	17	66
The Global HIV Prevention Coalition	2017	17	1	16
WHO SI^d^	2015	208	92	116

Indicator count refers to indicators as listed on original document.

^a^ Joint United Nations Programme on HIV/AIDS Global AIDS Monitoring

^b^ Global Fund HIV Monitoring & Evaluation Framework

^c^ U.S. President’s Emergency Plan for AIDS Relief Monitoring, Evaluation and Reporting Indicator Reference Guide

^d^ World Health Organization Consolidated Strategic Information Guidelines.

### 3.1 Indicator alignment between countries

The 21 NSPs (Table 3) contained 247 unique indicators. Of these, 162 (66%) were measured by only one country. The indicators with the most country‐level consensus were (in descending order of consensus):

‐ “%/# of general population who report testing in last 12 months and know test results” (13 countries),
‐ “%/# of general population who correctly identify main forms of HIV transmission and reject incorrect ones” (12 countries)
‐ “%/# of general population who had more than one partner in the last 12 months and report condom use at last sexual encounter” (11 countries)

**Table 3:** Number of countries’ national strategic plans (NSPs) that measure each unique HIV prevention indicator

Number of countries’ NSPs that measure each unique HIV prevention indicator	Number of indicators (%)
1	162 (66%)
2	41 (17%)
3	16 (6%)
4	6 (2.4%)
5	6 (2.4%)
6	2 (0.8%)
7	4 (1.6%)
8	3 (1.2%)
9	3 (1.2%)
10	1 (0.4%)
11	1 (0.4%)
12	1 (0.4%)
13+	1 (0.4%)
Total unique indicators collected by countries	247

The NSPs of the 11 high burden HIV countries of Eastern and Southern African included 158 indicators, with high variability between these countries; 107 (68%) of the indicators were listed in only one NSP. Of the 235 indicators measured by the 17 countries with high burden epidemics, 158 (67%) were measured by only one NSP, and the most commonly measured indicator (“%/# of general population who report testing in last 12 months and know test results”) was included in 11 NSPs. Of the 59 indicators measured by five countries with low HIV burdens, 42 (74%) were included in only one NSP and only 2 (3.5%) indicators were measured by four countries. Furthermore, only a weak positive correlation was found when comparing the number of indicators and HIV prevalence for each country (r = 0.30).

### 3.2 Indicator alignment between global stakeholders

The M&E guidance documents of the five global stakeholders (Table 4) included 89 unique indicators. Of these, 56 (62.9%) were included by only one stakeholder, seven were included by four stakeholders and none were present in all five documents. The seven indicators included by four stakeholders were (in alphabetical order):

‐ “%/# of general population who receive pre‐exposure prophylaxis (PrEP)”
‐ %/# of men who have sex with men (MSM) who report condom use during the last occurrence of anal sex”
‐ “%/# of MSM reached with programmes for HIV prevention”
‐ “%/# of people who inject drugs (PWID) reached with programmes for HIV prevention”
‐ “%/# of sex workers (SW) who report condom use with last client”
‐ “%/# of SW reached with programmes for HIV prevention”
‐ “# of syringes distributed per PWID”

**Table 4:** Number of global stakeholders that measure each unique HIV prevention indicator

Number of global stakeholders that measure each unique HIV prevention indicator	Number of indicators (%)
1	56 (63%)
2	11 (12%)
3	15 (17%)
4	7 (8%)
5	0 (0%)
Total unique indicators collected by global stakeholders	89

### 3.3 Indicator alignment between countries and global stakeholders

**Figure 1:**
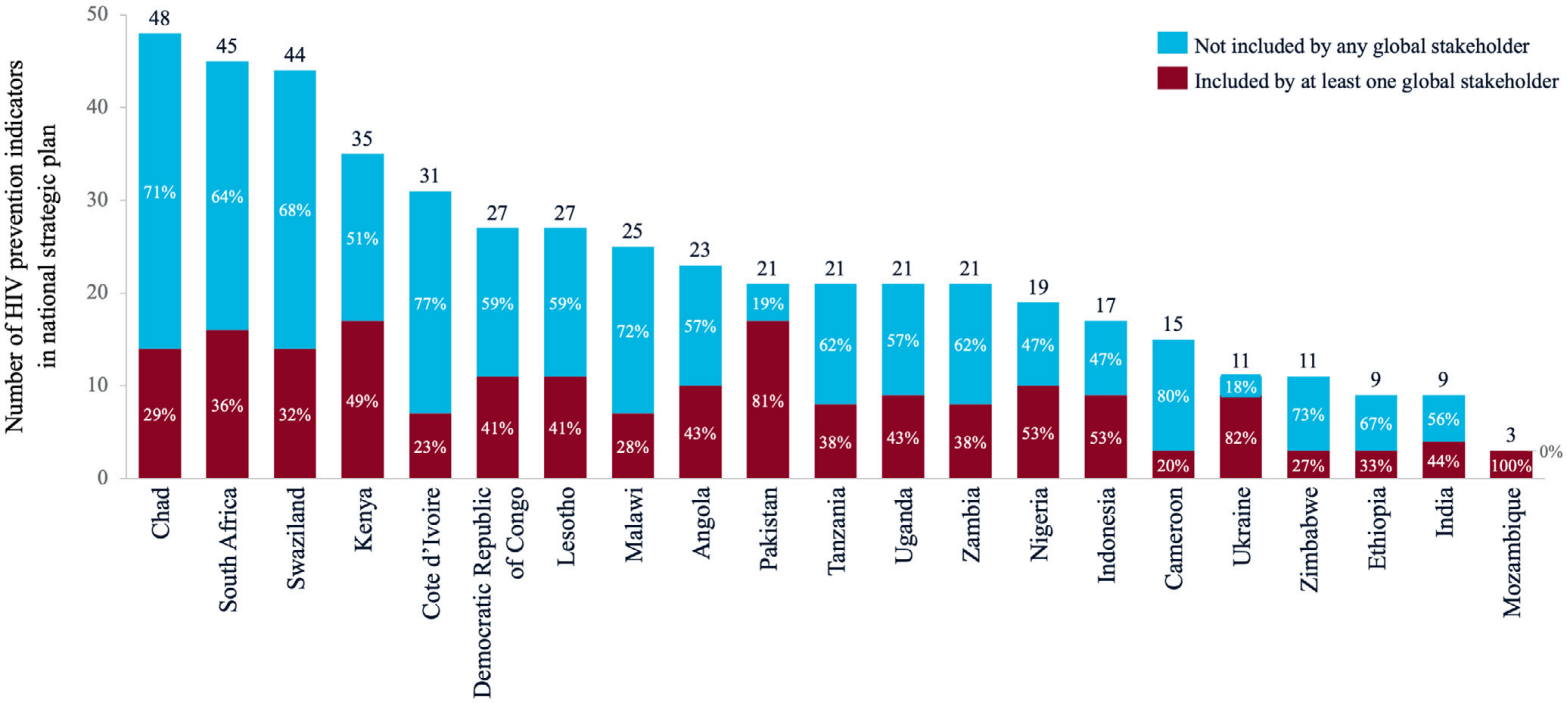
Number of HIV prevention indicators measured in the national strategic plan (NSP) of each country. Dark red bar indicates inclusion of that indicator in one or more global stakeholder documents.

Figure 1 shows the number of HIV prevention indicators included in NSPs within each country studied. The number of indicators collected ranged from 3 (Mozambique) to 48 (Chad). The proportion of indicators collected by each country that were included in any global stakeholder set ranged from 100% (Mozambique) to 20% (Cameroon). Averaging percentages across all countries, 56% of indicators collected at the country level did not appear in any of the evaluated global M&E documents. Of the 247 unique indicators collected by countries, 52 appeared in at least one global M&E document (21.1%) and 195 did not appear in any global document (78.9%).

**Figure 2:**
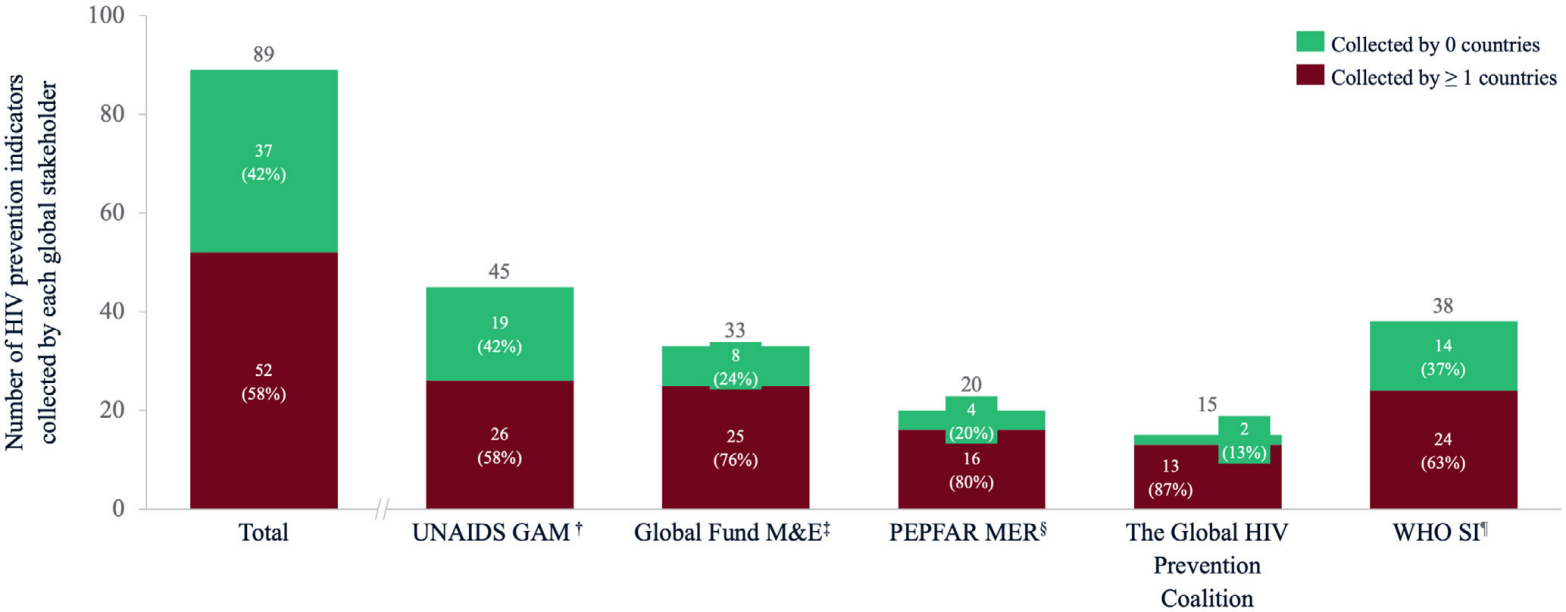
Number of HIV prevention indicators measured by five global stakeholders. Dark red bar indicates inclusion of that indicator in one or more countries’ National Strategic Plans. †Joint United Nations Programme on HIV/AIDS Global AIDS Monitoring. ^‡^Global Fund HIV Monitoring & Evaluation Framework. ^§^U.S. President’s Emergency Plan for AIDS Relief Monitoring, Evaluation, and Reporting Indicator Reference Guide. ^¶^World Health Organization Consolidated Strategic Information Guidelines.

The five global M&E documents (Figure 2) included 89 unique indicators. Of these, 52 (58.4%) were collected in at least one country, whereas 37 (41.6%) did not appear in any surveyed NSPs. By global stakeholder, the percentage of indicators not appearing in any NSP ranged from 13% (Global HIV Prevention Coalition) to 42% (GAM).

Tables S1 and S2 show global stakeholder indicators that were absent from any surveyed NSP and indicators measured by at least one surveyed NSP and at least one global stakeholder respectively. Examples of global stakeholder indicators absent from any surveyed NSP include:

‐ “%/# of general population diagnosed with gonorrhoea in past 12 months”
‐ “%/# of health units with PrEP services”

Examples of indicators measured by at least one surveyed NSP and at least one global stakeholder include:

‐ “%/# of MSM who report testing in the last 12 months and know test results”
‐ “%/# of PWID who report using sterilized equipment during last needle use”
‐ “%/# of males circumcised”

### 3.4 HIV prevention indicators by category

The categories with the most indicators were HIV education and outreach (17.6%), testing (17.3%), condom use (16.2%), stigma and discrimination (12.3%) and sexually transmitted infections (STIs) (7.0%). Among countries, the most frequently measured indicators by category were HIV education and outreach (19.0%), condom use (18.2%), testing (17.0%), stigma and discrimination (9.7%) and STIs (5.7%). Proportions of indicators in each category were similar by region and epidemic type. Among global stakeholders, the most frequently measured indicators by category were testing (27.0%), stigma and discrimination (14.6%), HIV education and outreach (13.5%), condom use (7.9%) and STIs (6.7%).

**Figure 3:**
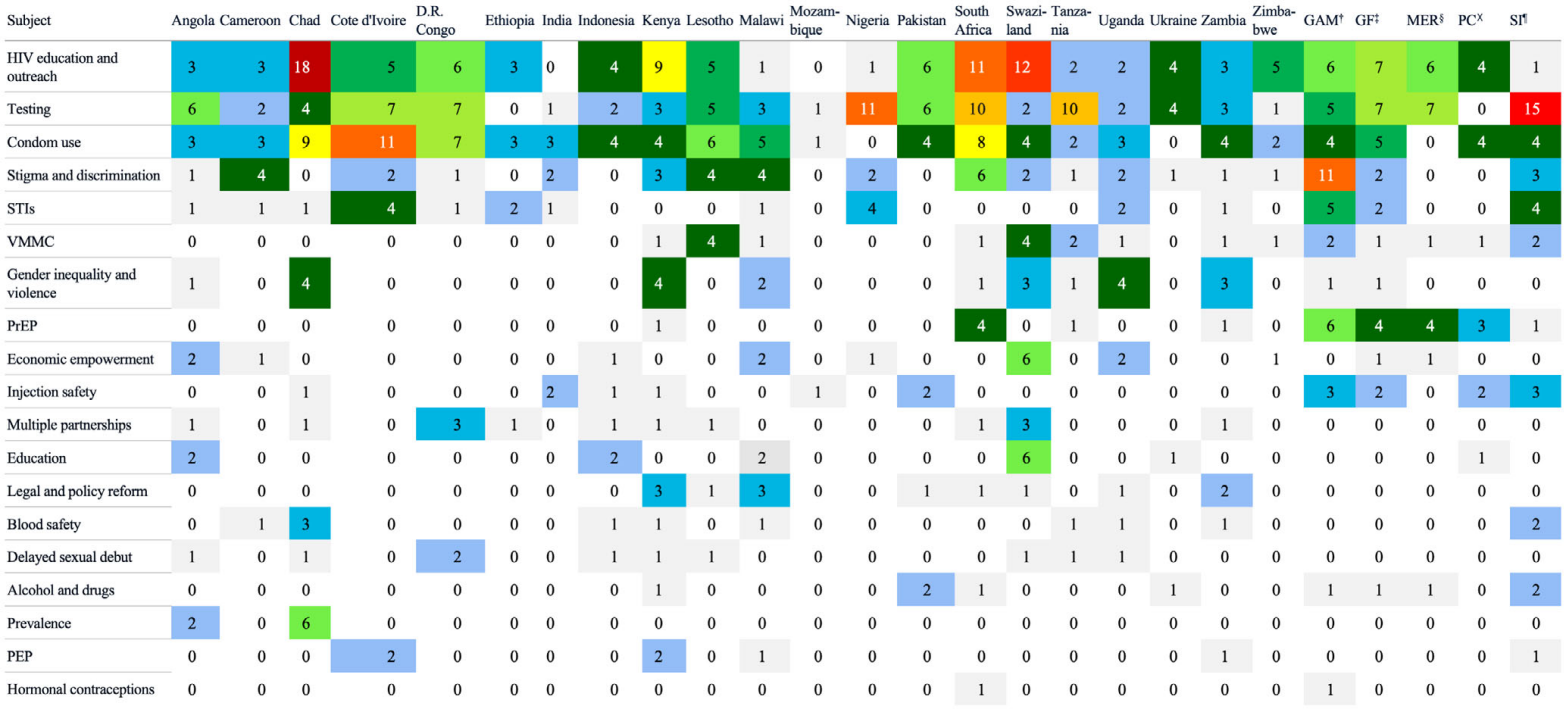
Number of HIV prevention indicators by subject collected by each country and global stakeholder. Number of indicators is represented by a heat map where dark red boxes indicate maximum number of indicators and white boxes indicate minimum number of indicators in National Strategic Plans. ^†^Joint United Nations Programme on HIV/AIDS Global AIDS Monitoring, ^‡^Global Fund HIV Monitoring & Evaluation Framework, ^§^U.S. President’s Emergency Plan for AIDS Relief Monitoring, Evaluation, and Reporting Indicator Reference Guide, X The Global HIV Prevention Coalition, ^¶^World Health Organization Consolidated Strategic Information Guidelines.

However, outside the top five categories, there was relatively little alignment in countries and global bodies in indicator type measured (Figure 3). For instance South Africa’s NSP and UNAIDS GAM were the only two documents that included hormonal contraceptives in any indicator. Likewise, four of the five global stakeholders (GAM, GF M&E, MER and SI) had indicators measuring alcohol and drug use, but only four countries (Kenya, Pakistan, South Africa, Ukraine) had an alcohol/drug‐related indicator. HIV education and outreach were the most commonly measured indicator category, with 90% (19/21) of countries (exceptions were India and Mozambique) and all five global stakeholders having at least one education/outreach indicator. Testing was the second most commonly measured indicator, with almost all countries and global stakeholders (exception: Ethiopia, Global HIV Prevention Coalition) having at least one testing indicator. Condom use was the third most commonly measured indicator category and there were four or more indicators measuring it in 11 countries; every surveyed country and global body had an indicator related to condom use, with the exception of two countries (Nigeria and Ukraine) and PEPFAR MER. There was high heterogeneity in the indicators’ specific language. For example, there were 20 unique ways to measure condom use amongst the surveyed NSPs and key global stakeholders. Furthermore, countries with low HIV burden (Democratic Republic of Congo, India, Indonesia, Pakistan and Ukraine) had, on average, 3.6 indicators on condom use and countries with high HIV burden had, on average, 4.3 indicators on condom use.

## 4 DISCUSSION

In order to understand the degree of consensus in HIV primary prevention measurement and evaluation, we reviewed the primary prevention indicators included in national strategic plans and global stakeholder M&E documents. Our review found limited agreement between global stakeholders and countries measurement of HIV prevention, with no indicators measured by more than 13 of the 21 countries, and no indicators measured by all five global stakeholders. There was a high range (from 3 to 48) in the number of indicators measured within each country and by global stakeholder (from 15 to 45). The number of indicators measured by each country was only weakly correlated with HIV rate by country. The majority (66%) of national indicators for HIV prevention were measured only by a single country; this heterogeneity amongst countries remained even when comparing countries with similar epidemics and/or geographies. Similarly, 63% of indicators in global stakeholder M&E documents was only measured by one global stakeholder. Additionally, there was a high degree of discordance between and within countries and global stakeholders, with 56% of country indicators not appearing in any of the evaluated global stakeholder M&E documents when averaged across countries. There was also a high degree of discordance in terms of prevention categories measured and the number of indicators measured in each category.

Our findings suggest that global stakeholders need to harmonize to a set of indicators that have a firm empiric basis, are strongly linked to HIV prevention outcomes, are harmonized across geographies to the extent possible, and meet the rigour of a framework (e.g. the UNAIDS indicator framework) [19]. Some degree of variability among global stakeholders, among countries, and between global stakeholders and countries is expected for several reasons. First, there is heterogeneity in terms of how global stakeholder documents are intended to be used. For example UNAIDS GAM is meant to be for annual global reporting on outcome and impact indicators, as defined by political declarations [10]. The Global HIV Prevention Coalition aims to monitor national prevention programmes [11], and MER indicators reflect US government priorities [9]. There is heterogeneity in reporting frequency (e.g. some MER indicators are reported quarterly, some semi‐annually and some annually). There are also differences in how often indicators are modified, with WHO SI guidelines updated every five years and Global Fund guidelines updated every three years, whereas MER indicators are updated yearly [8, 12]. This variability in purpose naturally affects the variability in indicators collected. This is true of NSPs as well. Kenya states that their NSP should be used to “track progress and continuously measure results” and for “enhancing transparency of all players,” whereas India’s NSP states that its aim is to “provide a solid framework to tailor the response to local needs based on context‐specific evidence.” Second, countries have different dynamics in their HIV epidemic and response, and that variability could be reflected in their indicators. For instance high burden countries may be focused on more general populations and low‐burden countries may focus more on key populations. There are substantial differences in the national HIV programmes and epidemiologic contexts of different countries. Third, indicators that are also measured by other programmes or sectors may not be reflected in HIV NSPs; for instance STI‐related indicators may be recorded in Sexual and Reproductive Health monitoring and evaluation plans. Finally, the process for creating NSPs and global stakeholder documents varies by country and organization, with some bodies implementing a more rigorous process than others, engaging a wide range of stakeholders and experts, whereas other processes are more ad‐hoc or political. Measuring prevention remains challenging because of the difficulty identifying high‐risk individuals, determining which prevention interventions are effective, and then taking those interventions to scale. Some variability in measurement is useful as it provides complementary perspectives on the state of HIV prevention, but attention is needed on where variability is expected and where measurement should be aligned.

Despite some of the potential explanations for the lack of consensus, the analysis suggests a general lack of global alignment on measurement priorities and approaches within HIV prevention [20]. For example indicators measured by four of five global stakeholders were primarily measurements of key populations or PrEP, whereas the indicators most measured by countries were on testing, condom use and HIV education in general populations. There does not seem to be a clear explanation for these differences, but does suggest discordance and underscores the lack of a unified global prevention response.

One example where variability was particularly stark was in indicators that measure condom use. For example, Cote d’Ivoire listed 11 out of 31 indicators in their NSP within in the single category of condom use, whereas Nigeria and Ukraine did not measure condom use at all. In addition, there was a high variability in the language used between different condom indicators at both the country and global levels. Another notable example was in the measurement of stigma and discrimination, which was one of the top five categories in both countries and global stakeholders, yet had significant heterogeneity and definitions used.

Heterogeneity in indicators and definitions can have real costs; for example resources are required to collect indicators; funding may be at risk if indicators are not collected. As an example, the Norwegian Refugee Council found that if their nine largest donors used the same financial reporting format, the organization would save 11,000 hours per year on financial reporting [21]. Frontline health workers at health facilities are often the most impacted by large numbers of indicators, as described in an assessment performed by the WHO [22]. There are also costs at the programmatic level in terms of the human and financial resources required to collect and report large numbers of indicators [21]. This is especially the case in the HIV response, in which there are multiple global stakeholders and large financial investments. Of note, indicators that focused solely on testing or treatment of people living with HIV were not included in this analysis; the reporting burden is broader than prevention indicators alone. Larger than necessary indicators sets, particularly those with varying definitions for similar indicators, are likely to result in poorer data collection and challenges in assessing performance at the national and global level.

National strategic plans and global stakeholder documents can serve as a proxy for a country or global body’s priorities, and some variability is expected and can be beneficial. Yet the degree of heterogeneity between selected indicators seems to indicate an opportunity to reevaluate and better understand the process for prioritizing and selecting indicators for HIV prevention at the national and global levels. The current UNAIDS‐led Monitoring Technical Advisory Group – the mandate of which is to harmonize national and global HIV indicators – should re‐evaluate and strengthen the process of selecting and prioritizing indicators for HIV prevention. Concomitantly, donors with reporting requirements should commit to prioritizing alignment, and leverage existing metrics whenever possible (e.g. through the use of the UNAIDS Indicator Repository) [23]. Greater alignment behind clearer and more consistent goals and measurements could ultimately catalyse action for more effective HIV prevention programmes.

## 5 LIMITATIONS

The potential limitations of this study include the availability and translation of surveyed NSPs. This analysis only includes 21 of 28 possible low‐/middle‐income 2015 Fast‐Track countries, although the included NSPs account for over 90% of the estimated burden of new infections among the 28 countries [18]. Additionally, we assume that all NSPs and stakeholders use similar definitions for key populations or programmes (e.g. that the definition of “sex workers” is consistent across countries and global organizations). Furthermore, as mentioned earlier, we anticipate that some of the differences may be related to the differential timing of the development of the NSPs and global stakeholder’s indicator sets, although we only included recent sets (2014 and onward) to mitigate this to the extent possible.

## 6 CONCLUSIONS

Reaching ambitious global 2030 goals for ending the HIV epidemic will require a more effective HIV prevention response. This analysis suggests that the measurement of HIV prevention programmes is highly heterogeneous and can reasonably be considered one of the factors impeding progress. The vast number of indicators and their diverse formats create a high burden of collection on national systems and reflects the lack of clarity about priorities for HIV prevention in various settings and the best indicators for measurement. If more effective HIV prevention programmes are to be developed, greater attention to creating unified frameworks for measurement will be an important part of the process.

## COMPETING INTERESTS

None declared.

## AUTHORS’ CONTRIBUTIONS

Sayuri Sekimitsu collected the data, performed the analysis and drafted the manuscript. Jacqueline DePasse, Jim Larson, Anna Carter and Charles Holmes provided guidance on the analysis, oversaw the study and provided substantial edits to the manuscript. Kristen Earle contributed to data collection and the design of the analysis. Kate Daley contributed to subanalyses. Brian Rice and Mary Mahy provided input throughout. Michelle Morrison and Geoff Garnett provided guidance on the analysis and input throughout. All authors discussed the results and contributed to the final manuscript.

## Supporting information


**Table S1.** Global stakeholder indicators that are absent from any surveyed national strategic plans (NSPs)
**Table S2.** Indicators measured by at least one surveyed national strategic plan (NSP) and at least one global stakeholder
