# Supplementary material for: High variability in the measurement of HIV primary prevention activities and outcomes
Source: J Int AIDS Soc. 2020 Dec 20;23(12):e25645. doi: 10.1002/jia2.25645 (PMC7750494; doi:10.1002/jia2.25645)
Supplement: Supplementary file 1 — Table S1. Global stakeholder indicators that are absent from any surveyed national strategic plans (NSPs) Table S2. Indicators measured by at least one surveyed national strategic plan (NSP) and at least one global stakeholder [file JIA2-23-e25645-s001.docx]

**Appendix Table S1. Global stakeholder indicators that are absent from any surveyed national strategic plans (NSPs)**

| **Indicator** | **GAM**^†^ | | **GF**^‡^ | | **MER**^§^ | | | **PC**^¶^ | | **SI^\|^** | |
| --- | --- | --- | --- | --- | --- | --- | --- | --- | --- | --- | --- |
| %/# of adolescent girls and young women reached with programs for HIV prevention |  | | √ | |  | | |  | |  | |
| %/# of adolescent girls and young women who underwent testing in last 12 months and know test results |  | | √ | |  | | |  | |  | |
| %/# of general population diagnosed with gonorrhoea in past 12 months | √ | |  | |  | | |  | | √ | |
| %/# of general population diagnosed with syphilis | √ | |  | |  | | |  | | √ | |
| %/# of general population who report urethral discharge in the past 12 months | √ | |  | |  | | |  | | √ | |
| %/# of general population who tested for HIV using a self-test kit |  | |  | |  | | |  | | √ | |
| %/# of general population who underwent retesting in last 12 months and know test results |  | |  | |  | | |  | | √ | |
| %/# of health units where all therapeutic injections are given with new, disposable single-use injection equipment |  | |  | |  | | |  | | √ | |
| %/# of health units with adequate blood transfusion requirements |  | |  | |  | | |  | | √ | |
| %/# of health units with no stock-outs of needles or syringes |  | |  | |  | | |  | | √ | |
| %/# of health units with pre-exposure prophylaxis services |  | |  | |  | | | √ | |  | |
| %/# of men who have sex with men who avoid health care because of stigma and discrimination | √ | |  | |  | | |  | |  | |
| %/# of men who have sex with men who have sex with men with active syphilis | √ | | √ | |  | | |  | |  | |
| %/# of men who have sex with men who suffered discrimination at healthcare facility | √ | |  | |  | | |  | |  | |
| %/# of Non-injecting drugs users who are still in treatment 6 months after starting opioid substitution therapy |  | | √ | |  | | |  | | √ | |
| %/# of orphans with partners who know their status |  | |  | | √ | | |  | |  | |
| %/# of people living with HIV who report seeing stigmatizing or discriminatory behaviour against people living with HIV |  | |  | |  | | |  | | √ | |
| %/# of people living with HIV who suffered discrimination at healthcare facility | √ | | √ | |  | | |  | |  | |
| %/# of pregnant/breastfeeding women who underwent testing for syphilis and received their test results within the last 12 months |  | | √ | |  | | |  | | √ | |
| %/# of priority populations reached with programs for HIV prevention |  | |  | | √ | | |  | |  | |
| %/# of prisoners who receive pre-exposure prophylaxis | | √ | |  | |  |  | |  | |  |
| %/# of prisoners who suffered discrimination at healthcare facility | | √ | |  | |  |  | |  | |  |
| %/# of prisoners who underwent retesting in last 12 months and know test results | |  | |  | |  |  | | √ | |  |
| %/# of people who inject drugs who avoid health care because of stigma and discrimination | | √ | |  | |  |  | |  | |  |
| %/# of people who inject drugs who report use of condom in last sexual intercourse | | √ | |  | |  |  | |  | |  |
| %/# of people who inject drugs who suffered discrimination at healthcare facility | | √ | |  | |  |  | |  | |  |
| %/# of sex workers diagnosed with syphilis | | √ | |  | |  |  | |  | |  |
| %/# of sex workers who avoid health care because of stigma and discrimination | | √ | |  | |  |  | |  | |  |
| %/# of sex workers who suffered discrimination at healthcare facility | | √ | |  | |  |  | |  | |  |
| %/# of transgender people who avoid health care because of stigma and discrimination | | √ | |  | |  |  | |  | |  |
| %/# of transgender people who receive pre-exposure prophylaxis | | √ | | √ | | √ |  | |  | |  |
| %/# of transgender people who report use of condom in last sexual intercourse | | √ | | √ | |  |  | |  | |  |
| %/# of transgender people who suffered discrimination at healthcare facility | | √ | |  | |  |  | |  | |  |
| %/# of transgender people who underwent retesting in last 12 months and know test results | |  | |  | |  |  | | √ | |  |
| %/# of transgender people who underwent testing and know results | |  | |  | | √ |  | |  | |  |
| %/# of vulnerable populations reached with programs for HIV prevention | |  | | √ | |  |  | |  | |  |
| Legal/policy provisions for pre-exposure prophylaxis introduced and drugs registered | |  | |  | |  | √ | |  | |  |

Legend of table: † Joint United Nations Programme on HIV/AIDS Global AIDS Monitoring ‡ Global Fund HIV Monitoring & Evaluation Framework, § U.S. President’s Emergency Plan for AIDS Relief Monitoring, Evaluation, and Reporting Indicator Reference Guide, ¶ Joint United Nations Programme on HIV/AIDS Global HIV Prevention Coalition Indicators, **|** World Health Organization Consolidated Strategic Information Guidelines

**Appendix Table S2. Indicators measured by at least one surveyed national strategic plan (NSP) and at least one global stakeholder**

| **Indicator** | **Number of NSPs** | **GAM**^†^ | **GF**^‡^ | **MER**^§^ | **PC**^¶^ | **SI^\|^** |
| --- | --- | --- | --- | --- | --- | --- |
| %/# of general population who underwent testing in last 12 months and know test results | 13 | √ | √ |  |  | √ |
| %/# of general population who correctly identify main forms of HIV transmission and reject incorrect ones | 12 | √ |  |  |  |  |
| %/# of general population who had more than one partner in the last 12 months and report condom use at last sexual encounter | 11 |  | √ |  | √ | √ |
| %/# of sex workers who report condom use with last client | 9 | √ | √ |  | √ | √ |
| %/# of sex workers reached with programs for HIV prevention | 9 | √ | √ | √ | √ |  |
| %/# of men who have sex with men who report condom use during last occurrence of anal sex | 8 | √ | √ |  | √ | √ |
| %/# of general population reached with programs for HIV prevention | 8 |  |  |  | √ |  |
| %/# of males who are circumcised | 7 | √ |  | √ | √ |  |
| %/# of transfused blood tested for HIV/Hepatitis/Syphilis | 7 |  |  |  |  | √ |
| %/# of sex workers who underwent testing in last 12 months and know test results | 7 | √ | √ |  |  | √ |
| %/# of men who have sex with men reached with programs for HIV prevention | 7 | √ | √ | √ | √ |  |
| %/# of orphans living in households receiving basic support to care | 6 |  | √ |  |  |  |
| %/# of general population who underwent testing and know results | 6 |  |  | √ |  |  |
| %/# of people who inject drugs who report use of condom in last sexual intercourse | 5 |  | √ |  |  | √ |
| %/# of men who have sex with men who underwent testing in last 12 months and know test results | 5 | √ | √ |  |  | √ |
| %/# of general population who suffered physical/sexual violence by intimate partner in past 12 months | 4 | √ | √ |  |  |  |
| %/# of general population who report condom use during at-risk sex | 4 | √ |  |  |  |  |
| %/# of people who inject drugs who report using sterilized equipment during last needle use | 4 | √ | √ |  | √ |  |
| %/# of people who inject drugs on opioid substitution therapy | 4 | √ |  | √ |  | √ |
| %/# of prisoners reached with programs for HIV prevention | 3 | √ |  | √ |  |  |
| %/# of sex workers who underwent testing and know results | 3 |  |  | √ |  |  |
| %/# of people who inject drugs who underwent testing and know results | 3 |  |  | √ |  |  |
| %/# of orphans who receive financial support | 3 |  |  | √ |  |  |
| %/# of people who inject drugs who underwent testing in last 12 months and know test results | 3 | √ | √ |  |  | √ |
| %/# of people who inject drugs reached with programs for HIV prevention | 3 | √ | √ | √ | √ |  |
| %/# of health units with adequate service | 3 |  |  |  |  | √ |
| %/# of syringes distributed | 2 | √ | √ |  | √ | √ |
| %/# of men who have sex with men who underwent testing and know results | 2 |  |  | √ |  |  |
| %/# of general population who report discriminatory attitudes towards people living with HIV/AIDS | 2 | √ | √ |  |  | √ |
| %/# of medical male circumcisions performed | 2 | √ | √ |  |  | √ |
| %/# of general population who receive pre-exposure prophylaxis | 2 | √ |  | √ | √ | √ |
| %/# of male and female condoms distributed | 2 |  |  |  | √ |  |
| %/# of general population reached with programs for HIV prevention in school | 2 |  | √ |  |  |  |
| %/# of prisoners who underwent testing in last 12 months and know test results | 2 |  |  |  |  | √ |
| %/# of health units with HIV services | 2 |  |  |  |  | √ |
| %/# of health units with stock out of HIV test kits within the last 3 months | 2 |  |  |  |  | √ |
| %/# of men who have sex with men who receive pre-exposure prophylaxis | 1 | √ | √ | √ |  |  |
| %/# of vulnerable populations who underwent testing in last 12 months and know test results | 1 |  | √ |  |  |  |
| %/# of general population for whom modern contraceptives are available | 1 | √ |  |  |  |  |
| %/# of sex workers who receive pre-exposure prophylaxis | 1 | √ | √ | √ |  |  |
| %/# of people who inject drugs who receive pre-exposure prophylaxis | 1 | √ |  |  |  |  |
| %/# of transgender people reached with programs for HIV prevention | 1 | √ | √ | √ |  |  |
| %/# of transgender people who underwent testing in last 12 months and know test results | 1 | √ | √ |  |  | √ |
| %/# of key populations who experience discrimination by healthcare workers | 1 |  |  |  |  | √ |
| %/# of health units with post-exposure prophylaxis services | 1 |  |  |  |  | √ |
| %/# of sex workers who underwent retesting in last 12 months and know test results | 1 |  |  |  |  | √ |
| %/# of men who have sex with men who underwent retesting in last 12 months and know test results | 1 |  |  |  |  | √ |
| %/# of people who inject drugs who underwent retesting in last 12 months and know test results | 1 |  |  |  |  | √ |
| %/# of adolescent girls and young women who receive pre-exposure prophylaxis | 1 |  | √ |  |  |  |
| %/# of prisoners who underwent testing and know results | 1 |  |  | √ |  |  |
| %/# of general population who complete secondary school | 1 |  |  |  | √ |  |
| %/# of general population who experience moderate or severe adverse events during or following voluntary male medical circumcision surgery | 1 |  |  |  |  | √ |

Legend of table: † Joint United Nations Programme on HIV/AIDS Global AIDS Monitoring ‡ Global Fund HIV Monitoring & Evaluation Framework, § U.S. President’s Emergency Plan for AIDS Relief Monitoring, Evaluation, and Reporting Indicator Reference Guide, ¶ Joint United Nations Programme on HIV/AIDS Global HIV Prevention Coalition Indicators, **|** World Health Organization Consolidated Strategic Information Guidelines
